# The Role of Taurine in Mitochondria Health: More Than Just an Antioxidant

**DOI:** 10.3390/molecules26164913

**Published:** 2021-08-13

**Authors:** Chian Ju Jong, Priyanka Sandal, Stephen W. Schaffer

**Affiliations:** 1 Neuroscience and Pharmacology, Carver College of Medicine, University of Iowa, Iowa City, IA 52242, USA; priyanka-sandal@uiowa.edu; 2 Department of Pharmacology, College of Medicine, University of South Alabama, Mobile, AL 36688, USA; sschaffe@southalabama.edu

**Keywords:** taurine, mitochondria, antioxidant, 5-taurinomethyluridine, oxidative stress, apoptosis

## Abstract

Taurine is a naturally occurring sulfur-containing amino acid that is found abundantly in excitatory tissues, such as the heart, brain, retina and skeletal muscles. Taurine was first isolated in the 1800s, but not much was known about this molecule until the 1990s. In 1985, taurine was first approved as the treatment among heart failure patients in Japan. Accumulating studies have shown that taurine supplementation also protects against pathologies associated with mitochondrial defects, such as aging, mitochondrial diseases, metabolic syndrome, cancer, cardiovascular diseases and neurological disorders. In this review, we will provide a general overview on the mitochondria biology and the consequence of mitochondrial defects in pathologies. Then, we will discuss the antioxidant action of taurine, particularly in relation to the maintenance of mitochondria function. We will also describe several reported studies on the current use of taurine supplementation in several mitochondria-associated pathologies in humans.

## 1. Introduction

Mitochondrial dysfunction, along with oxidative stress, is a key hallmark of various pathologies, such as aging [1,2], cardiovascular diseases [3,4], mitochondrial diseases [5,6], metabolic syndrome [7,8], cancer [9,10] and neurological disorders, such as neurodegenerative diseases [11,12] and neurodevelopmental disorders [13,14]. Often, antioxidant therapy, such as coenzyme Q [15], mitoQ [16,17], vitamin E [18], gingko biloba extracts [19], ebselen [20], creatine [21], lipoic acid [22], melatonin [23,24] and l-arginine [25,26], provide some protections, potentially by improving the mitochondrial function and reducing oxidative stress in these diseases. Recently, taurine, a sulfur-containing amino acid, has been approved in Japan in treating stroke-like episodes in patients with mitochondrial myopathy, encephalopathy, lactic acidosis and stroke-like episodes (MELAS), which is a mitochondrial disease [27,28]. Indeed, the use of taurine dates back to 1985, as taurine was first used to treat patients with congestive heart failure in Japan [29,30]. In addition, taurine supplementation has been shown to improve the exercise capacity of patients with heart failure [31], which is likely due to improvement of the myocardial energy production. Although taurine was first identified in the 1800s [32], the mitochondrial actions of taurine still remain unclear and underappreciated. This review, therefore, will provide an overview of the significant role of taurine in the maintenance of mitochondrial function. Clinical studies using taurine therapy in mitochondria-targeted pathologies will also be discussed.

## 2. Mitochondria Biology

Mitochondria are cellular organelles that regulate various essential cellular processes [33,34,35,36,37]. The mitochondria consist of two membranes, an ion impermeable inner membrane and a permeable outer membrane, which envelopes a soluble matrix containing cristae [38]. There are hundreds of mitochondria in one cell and each mitochondrion contains 2–10 copies of mitochondrial DNA (mtDNA) [39]. The mtDNA encodes 13 polypeptides, which are components of the electron transport chain, as well as two ribosomal RNAs (rRNA) and 22 transfer RNAs (tRNA), which regulate the synthesis of mitochondrial proteins [40]. Predominantly known as the powerhouse of the cell, mitochondria provide cellular energy by generating ATP via oxidative phosphorylation. Reducing equivalents, such as NADH and FADH_2_, produced via the tricarboxylic acid (TCA) cycle, deliver electrons along the electron transport chain to reduce molecular oxygen to water. The influx of electrons along the electron transport chain creates a transmembrane proton gradient that drives ATP synthesis via the ATP synthase (F_o_F_1_ complex synthase) [41]. A consequence of electron transport along the electron transport chain is the generation of reactive oxygen species (ROS), where one electron reduces molecular oxygen to produce a superoxide anion (O_2_^−·^) [42]. When catalyzed by the antioxidant superoxide dismutase (SOD), O_2_^−·^ is dismutated to hydrogen peroxide (H_2_O_2_) and molecular oxygen. H_2_O_2_ can be further partially reduced to a hydroxyl radical (OH^−^), which is a highly reactive species [42].

The mitochondria are the main source of superoxide production, primarily via Complex I and Complex III of the electron transport chain [43,44]. Under normal conditions, 2% of electrons are diverted to reduce molecular oxygen to produce a superoxide [42]. Physiologically, ROS has been shown to regulate various crucial cellular processes, such as cellular differentiation [45,46], autophagy [47,48], metabolic adaptation [49,50] and immune cell activation [50,51,52]. Pathologically, ROS has often been shown to cause harm to cells, which are described as follow. One, ROS is capable of inducing mitochondrial and nuclear DNA damage [53,54]. Two, ROS causes irreversible protein oxidation. ROS oxidizes the side chain of four key amino acids, lysine, arginine, proline and threonine, which adds ketone or aldehyde groups to proteins and alters the protein structure and function [55,56]. Three, ROS oxidizes cellular and organelle membranes, which consist of polyunsaturated fatty acids [57]. Cardiolipin is a unique phospholipid localized in the inner membrane of the mitochondria as it contains a polar head group that traps protons for oxidative phosphorylation [58], three glycerol backbones and four acyl chains [59]. A phospholipid generally consists of a polar headgroup, a glycerol backbone and hydrophobic acyl chains [60]. There has been increasing evidence showing cardiolipin being crucial for the functionality of the mitochondria. Primarily, cardiolipin maintains the structural integrity of the mitochondrial membranes [61,62], as well as stability and proper functioning of proteins and enzyme complexes involved in oxidative phosphorylation [63,64,65]. Oxidation of cardiolipin induces mitochondrial dysfunction, as has been shown in several in vitro studies. These studies have shown impaired cellular metabolism and a sluggish activity of the electron transport chain [64,66,67], as well as enhanced cell death, as evidenced by mitochondrial permeability transport pore opening and cytochrome c release [68,69]. To counteract excessive ROS production, the cell contains an antioxidant defense system, which encompasses enzymatic antioxidants such as mitochondria-localized manganese superoxide dismutase (MnSOD), cytosolic-localized zinc SOD (ZnSOD) and copper SOD (CuSOD), catalase and glutathione peroxidase. MnSOD, ZnSOD and CuSOD catalyze the dismutation of O_2_^−^ into water and H_2_O_2_ [42]. Both catalase and glutathione peroxidase break down H_2_O_2_ into water and oxygen [42,70]. When ROS is excessively produced, it can overwhelm the antioxidant defenses and this causes oxidative stress [71,72]. Often, increased oxidative stress further exacerbates cellular damage [73,74].

## 3. Mitochondria in Pathologies

Impairment of mitochondrial function has been commonly reported in pathologies such as aging, cardiovascular diseases, mitochondrial diseases, metabolic syndrome, cancer and neurological disorders [1,2,3,4,5,6,7,9,10,11,13,14,75]. Often, these pathologies are characterized by increased ROS production [1,2], reduced ATP generation [5,7], apoptosis [75,76], impaired mitochondrial biogenesis [4,76], impaired activity of electron transport chain [3,7,77] and mitochondrial calcium mishandling [4]. Recently, increasing evidence from in vitro [78,79,80,81,82,83,84] and in vivo [85,86,87,88,89] studies has demonstrated the beneficial effects of taurine in maintaining mitochondrial functions.

## 4. Taurine Biology

**Figure 1:**
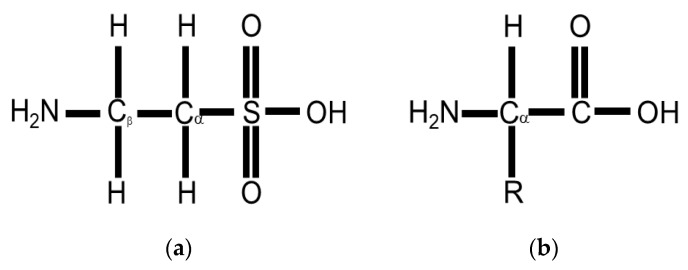
(**a**) Taurine or 2-aminoethane-sulfonic acid is a β-sulfonic amino acid as it has a sulfonyl group rather than a carboxyl group attached to the alpha carbon and an amino group on the beta carbon; (**b**) a standard amino acid contains an alpha carbon, to which an both an amino group and a carboxyl group are attached.

**Figure 2:**
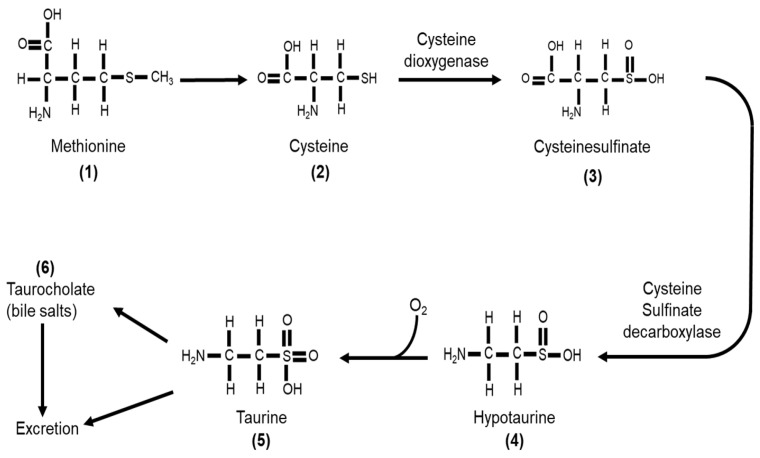
Taurine (**5**) is synthesized from either methionine (**1**) or cysteine (**2**). Cysteine dioxygenase catalyzes the conversion of cysteine (**2**) to cysteinesulfinate (**3**), which then is converted to hypotaurine (**4**) by cysteine sulfinate decarboxylase. Hypotaurine (**4**) is readily oxidized to form taurine (**5**), which can be excreted directly or as a conjugate with bile acids such as taurocholate (**6**).

Taurine or 2-aminoethane-sulfonic acid is a unique amino acid as it has a sulfonyl group on the C-terminus and an amino group residing on the β-carbon (Figure 1a) rather than α-carbon (Figure 1b) [90]. Taurine, therefore, is a β-sulfonic amino acid (Figure 1). Taurine was first identified by Tiedemann and Gmelin, who isolated taurine in 1827 from the bile of the ox, *Bos taurus* [32]. As described in Figure 2, taurine (**5**) is synthesized in the liver from methionine (**1**) or cysteine (**2**) to produce hypotaurine (**4**) by cysteine dioxygenase and cysteine sulfonic acid decarboxylase (CSAD). Cysteine dioxygenase converts methionine or cysteine (**1**–**2**) to cysteinesulfinate (**3**), while CSAD converts cysteine sulfinate (**3**) to hypotaurine (**4**). Hypotaurine (**4**) is then readily oxidized to taurine (5), which may be excreted directly or as a conjugate with bile salts such as taurocholate (**6**) [90].

In most mammals including humans, rodents and some primates, taurine is considered a conditionally essential amino acid, as cysteine dioxygenase and CSAD are present abundantly. Mammals primarily depend on taurine biosynthesis in vivo [91] and partially from diet, such as from meat, seafood and human milk [92,93,94]. Newborns and young infants are unable to synthesize taurine as well as adult humans, and therefore, are dependent on a taurine-supplemented diet [95]. Clinical studies investigating infants supplemented with (30–40 μM/dL) or without taurine showed that inadequate taurine supplementation impairs lipid absorption and bile acid secretion and causes hepatic and retinal dysfunction [96,97,98,99]. Due to the importance of taurine in neonatal development, mothers are strongly encouraged to breastfeed, due to the high concentration of taurine in breast milk, or feed infants with taurine-supplemented formulas and taurine-supplemented total parenteral nutrition [97,99,100]. On the other hand, the activities of taurine biosynthetic enzymes are low in cats, dogs and foxes, and therefore they primarily depend on a taurine-supplemented diet [101,102]. When fed with a diet deficient in taurine, these animals developed pathologies such as cardiomyopathy and myocardial dysfunction [101,103,104], retinal and tapetal degeneration that leads to blindness [105,106,107], neurological abnormalities [108,109], weakened immune response [110], pregnancy and fetal development complications [111,112], as well as gastrointestinal problems [113,114]. In contrast, when fed with taurine-supplemented diets, these animals were protected against pathologies such as cardiomyopathy [115,116], seizure [108,117], and retinopathy [118], and showed improved reproductive performance and neurological development [119]. Taurine has also been added to energy drinks such as Red Bull, Monster, Tab Energy and Rockstar [120]. It was estimated that on average, there is 750 mg of taurine in an 8 oz can of energy drink [120]. While energy drinks mainly provide an energy boost, the exact role of taurine in energy drinks remains unclear as energy drinks also contain additional additives such as caffeine, ginseng, vitamins, antioxidants and sugars [121]. However, two reviews by Kurtz et al. [122] and Seidel et al. [123] have described the influence of taurine on exercise performance.

Taurine is ubiquitously expressed in most tissues, particularly in the excitable tissues such as the heart, retina, brain and muscles [32]. The intracellular concentration of taurine is commonly 5–50 mM and the plasma concentration of taurine is approximately 100 μM. When taurine is supplemented, the plasma taurine content usually reaches its peak within 1 h to 2.5 h of taurine intake [124,125]. Ghandforoush-Sattari et al. [124] conducted an analysis on the pharmacokinetics of oral taurine supplementation (4 g) in healthy adults. These individuals, who had fasted overnight, showed a baseline taurine content in a range of 30 μmol to 60 μmol. Then, 1.5 h after taurine intake, the plasma taurine content increased to approximately 500 μmol. Plasma taurine content subsequently decreased to baseline level 6.5 h after taurine intake. This study would be consistent with the common notion that excess plasma taurine is mostly excreted via urine or being transported to tissues. As taurine is only synthesized in the liver, maintenance of a high concentration of taurine in other tissues depends on the taurine uptake from the blood via a sodium-dependent taurine transporter (*TauT*). This *TauT* has a higher affinity for β-amino acids, such as taurine, but a lower affinity for α-amino acids [90]. The importance of this taurine transporter has been evidenced in mouse models lacking the *TauT* gene [126,127], as well as in in vivo and in vitro studies utilizing taurine transporter competitive inhibitors, such as β-alanine [128,129] or guanidinoethanesulfonate (GES) [130,131].

Two mouse models lacking the *TauT* (TauTKO) were generated by Ito’s group [126] and Warskulat’s group [127]. Both TauTKO mouse models had reduced taurine concentrations in the heart, skeletal muscles and retina, validating the requirement of the taurine transport from the liver to these tissues [126,127]. As a consequence of taurine deficiency, these TauTKO mice developed retinal degeneration, chronic liver disease, muscle atrophy, a decrease in exercise capacity and increased susceptibility to streptozotocin-induced diabetic nephropathy [126,127,132,133,134]. The TauTKO mice developed by the Ito’s group also showed evidence of cardiomyopathy, as indicated by diminished fractional shortening; ventricular remodeling, as shown by dilated ventricles; and reductions in ventricular wall thickness, as well as increased expression of fetal genes that serve as heart failure markers, such as atrial natriuretic peptide (ANP), brain natriuretic peptide (BNP) and β-myosin heavy chain (MHC) [126]. Further examination of the TauTKO hearts revealed mitochondrial swelling and disruption of the outer mitochondrial membrane, as well as a reduction in the activity of succinate dehydrogenase (SDH), which is a marker of mitochondrial enzyme [126]. In addition, TauTKO hearts contained defective mitochondria, as evidenced by smaller mitochondria size, impaired activities of the electron transport chain, oxidative stress and apoptosis [135]. The TauTKO hearts were also associated with impaired autophagy, which is the cellular quality control in degrading damaged proteins or organelles [136,137]. Defective mitochondria and impaired autophagy in TauTKO hearts, therefore, may contribute to the underlying development of cardiomyopathy in TauTKO mice. In addition, the same TauTKO mice showed premature aging, as characterized by shortened lifespan and acceleration of skeletal muscle senescence [138]. Interestingly, while the TauTKO mice developed by the Warskulat’s group showed normal cardiac function, the expression of fetal genes such as ANP, BNP and CARP (cardiac ankyrin repeat protein) increased in the TauTKO hearts, suggesting taurine depletion may predispose the mice to the development of a heart failure [127]. Based on the studies from these mouse models that lacked the *TauT*, it is convincing that taurine indeed has multiple physiological roles that include maintaining mitochondrial function.

Similarly, pharmacological inhibition of taurine transport using β-alanine or GES in both in vitro and in vivo studies resulted in significant pathological conditions, which include atrophic cardiac remodeling [126,139,140], oxidative stress [141,142], increased apoptosis [143], mitochondrial defects [83,84] and altered cardiac cell morphology [144], as well as loss of retinal ganglion cells that lead to retinopathy [128]. All these studies clearly demonstrated the importance of taurine as a cytoprotective agent with multiple physiological functions. Recently, increasing studies have focused on the antioxidant role of taurine in maintaining mitochondrial function.

## 5. Taurine as a Therapeutic Agent in Mitochondrial Dysfunction

**Figure 3:**
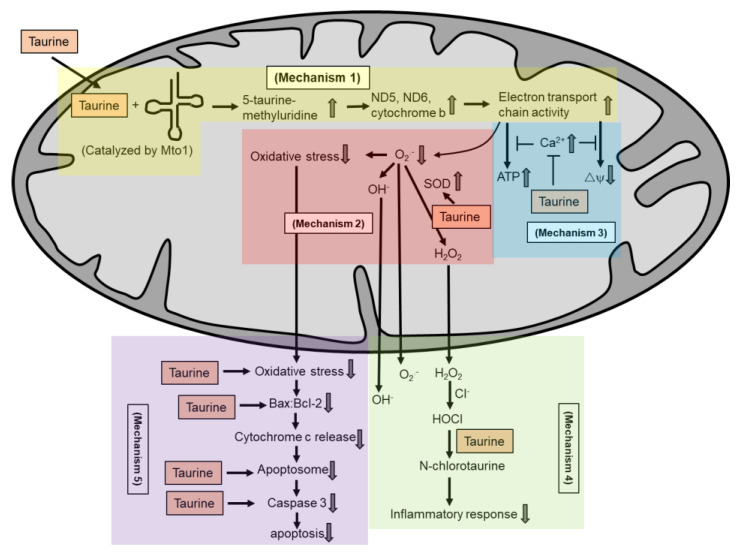
Taurine is known not as a radical scavenger. Several potential mechanisms by which taurine exerts its antioxidant activity in maintaining mitochondria health include: taurine conjugates with uridine on mitochondrial tRNA to form a 5-taurinomethyluridine for proper synthesis of mitochondrial proteins (mechanism 1), which regulates the stability and functionality of respiratory chain complexes; taurine reduces superoxide generation by enhancing the activity of intracellular antioxidants (mechanism 2); taurine prevents calcium overload and prevents reduction in energy production and the collapse of mitochondrial membrane potential (mechanism 3); taurine directly scavenges HOCl to form N-chlorotaurine in inhibiting a pro-inflammatory response (mechanism 4); and taurine inhibits mitochondria-mediated apoptosis by preventing caspase activation or by restoring the Bax/Bcl-2 ratio and preventing Bax translocation to the mitochondria to promote apoptosis (mechanism 5).

Various in vitro and in vivo studies have reported that taurine supplementation protects against mitochondrial dysfunction. Homma et al. [82] recently showed that taurine protects against metabolic impairment and mitochondrial dysfunction in MELAS patient-derived undifferentiated induced pluripotent stem cells (iPSCs) and the iPSC-derived retinal pigment epithelium (RPE). In a clinical study on oral taurine supplementation among MELAS patients, Ohsawa et al. [27] reported that taurine reduces the incidence of stroke-like episodes and increases the taurine modification of mitochondrial tRNA^Leu(UUR)^. Shetewy et al. [83] showed that taurine pretreatment protects against mitochondria damage and mitochondria fission in beta-alanine-treated mouse embryonic fibroblasts. Jong et al. [84] also showed that taurine pretreatment protects against the effect of beta-alanine-mediated taurine depletion on the opening of the mitochondrial permeability transition pore and subsequently inhibited apoptosis. In rat cardiomyocytes, taurine supplementation inhibits glucose-deprivation-induced mitochondrial oxidative stress, mitochondrial dysfunction, apoptosis and ER stress [78]. The aforementioned studies are some examples describing the protective roles of taurine in maintaining mitochondria health. In the following sections, we describe several mechanisms by which taurine may regulate mitochondrial health. All these mechanisms are summarized in Figure 3.

### 5.1. Taurine Forms a Complex with Mitochondrial tRNA

Taurine is primarily a free amino acid, although it does conjugate with bile acids to form taurocholate [90]. In 2002, a group of Japanese scientists discovered that taurine is a component of the mitochondrial tRNAs [145]. Specifically, they identified two taurine-containing modified uridines, namely 5-taurinomethyluridine (τm^5^u) and 5-taurinomethyl-2-thiouridine (τm^5^s^2^u). These conjugates are linked to the role of taurine as an antioxidant. Taurine conjugates with uridine at the anticodon wobble position of mitochondrial tRNA^Leu(UUR)^ or mitochondrial tRNA^Lys^ to form τm^5^u and τm^5^s^2^u, respectively [145,146]. These conjugation reactions, which are catalyzed by the mitochondrial optimization 1 (Mto1) in mammals [147], are required for precise anticodon–codon interactions for proper synthesis of mitochondrial-encoded proteins [148].

Based on the wobble hypothesis, the nucleoside at the first position of the anticodon forms hydrogen bonds with the third nucleoside of the codon, forming a wobble base pair. Normally, uridine at the anticodon wobble position base pairs with either adenine (A) or guanine (G) at the codon position of mRNA, which translates for leucine codons (UUA and UUG). An unmodified uridine at the anticodon wobble position can base pair with all four bases, A, G, cytosine (C) and uracil (U) at the third position of the codon [146]. These pairings subsequently result in mistranslation, as it also translates for phenylalanine codons (UUC and UUU) in addition to the usual leucine codons. However, when taurine conjugates with uridine at the anticodon wobble position, the taurine-modified uridine base pairs only with either adenine or guanine of the corresponding codons and translates for leucine codons (UUA and UUG) [145,148,149]. This proper anticodon–codon interaction is significantly important for proper expression of mitochondrial-encoded proteins [149,150], implicating the significance of post-transcriptional modification of uridine through taurine conjugation at the anticodon wobble position of mitochondrial tRNA^Leu(UUR)^. The importance of post-transcriptional modifications in tRNAs lies not only in ensuring proper codon recognition for translation accuracy, they are also essential in improving the efficiency of tRNA and facilitating the codon recognition by elongation factors or aminoacyl-tRNA synthetases. Consequently, a mutation in the post-transcriptional modification of mitochondrial tRNA^Leu(UUR)^ can affect protein synthesis as it can decrease RNase P processing, tRNA stability and aminoacylation, resulting in an abnormal tRNA conformation [150].

A defect in taurine conjugation of mitochondrial tRNA^Leu(UUR)^, which prevents the formation of 5-taurinomethyl-uridine, has been implicated in mitochondrial myopathy, encephalopathy, lactic acidosis and stroke-like episodes (MELAS), as well as myoclonus epilepsy associated with ragged-red fibers (MERRF) [82,145,146,148,149]. Indeed, an early study by Kirino et al. [149] showed that unmodified uridine of mitochondrial tRNA^Leu(UUR)^ weakens the binding affinity for the UUG codon, which could result in inefficient synthesis of mitochondrial proteins. Among the 13 polypeptides that encode mitochondrial proteins, ND5, ND6 and cytochrome b contain two, eight and two UUG codons, respectively. A deficiency in taurine conjugation of mitochondrial tRNA^Leu(UUR)^, therefore, may affect the synthesis of mitochondrial proteins. Indeed, Jong et al. [129] have shown a reduction in the protein levels of ND5 and ND6 in beta-alanine-treated cells. Both ND5 and ND6 are components of the complex I of the electron transport chain. A reduction in the expression of mitochondrial proteins ND5 and ND6 then cause the instability of the complex I, which could cause a sluggish transport of electrons across the respiratory chain, as well as diversion of electrons to oxygen to form a superoxide. Excessive superoxide production then can promote oxidative stress and overwhelm the antioxidant defenses [84,129]. Indeed, a recent study by Fakruddin et al. [147] showed that defective taurine-containing mitochondrial tRNA modification causes mitochondrial dysfunction and disrupts global protein homeostasis, thereby suggesting the significance of taurine-containing uridine modification in regulating global protein homeostasis. When taurine was supplemented, Homma et al. [82] observed an increase in the taurine modification of the mitochondrial tRNA^Leu(UUR)^, as well as an improvement in the mitochondrial function in the iPSC generated from a MELAS patient. While many other studies [28,78,81,82,83,84,88,89,93,99,117,145] have shown that taurine supplementation protects against mitochondrial dysfunction without definite underlying mechanisms, it is likely that the antioxidant function of taurine is associated with its role in the conjugation reaction with the uridine of the mitochondrial tRNA^Leu(UUR)^. However, this matter will require further validation.

### 5.2. Taurine Reduces Superoxide Generation in the Mitochondria

There have been many studies showing taurine as an antioxidant with a role in protecting against oxidative stress in the mitochondria [27,28,78,81,82,83,84,88,89,93,99,117,136,145]. However, the underlying mechanism by which taurine protects against oxidative stress in the mitochondria remains unclear, as Aruoma et al. [151] showed that taurine is not a radical scavenger. It is important to note that while taurine is incapable of scavenging classical ROS, taurine is a direct scavenger of hypochlorous acid (HOCl), which is generated from hydrogen peroxide (H_2_O_2_) in the presence of chloride ions, producing N-chlorotaurine [151,152]. The role of N-chlorotaurine is mainly in regulating the inflammatory response. Specifically, N-chlorotaurine has been shown to activate nuclear factor (erythroid-derived 2)-like 2 (Nrf2), which is a transcription factor that controls the transcription of various antioxidant genes and subsequently prevents inflammation [153,154,155].

While recent studies have suggested that the prevention of superoxide in the mitochondria is linked to the taurine conjugation of the mitochondrial tRNA [28,82,84,147], several studies have shown that taurine may exert its antioxidant function via different mechanisms. In germ cells, taurine has been shown to protect against oxidative stress by promoting the activity of Cu/Zn SOD [156]. Cu/Zn SOD is localized in the mitochondrial intermembrane space activity [157,158]. Indeed, taurine increases the protein levels but not mRNA levels of Cu/Zn SOD, suggesting that taurine mediates the effects of Cu/Zn SOD at the protein level. In another study by Tabassum et al. [159], the antioxidant role of taurine was attributed to the enhancement of intracellular reduced glutathione (GSH). GSH is essential for detoxification of xenobiotics, where GSH is oxidized to GSSG (oxidized glutathione) during oxidative stress. In a tamoxifen-treated liver, there is a reduction in the GSH levels, which increases the susceptibility to oxidative stress due to impaired antioxidant defense system. When tamoxifen-treated mice were co-treated with taurine, the GSH level was restored and oxidative stress was prevented. These findings suggest the physiological role of taurine in stabilizing the intracellular GSH levels. Other earlier studies by Pasantes et al. [160,161,162] have also suggested that taurine protects lipid membranes from tamoxifen-induced oxidative damage by acting as a membrane stabilizer, rather than directly acting against the oxidants. Indeed, a review by Hansen et al. [163] described the role of taurine as a buffer in the mitochondria matrix to stabilize the pH gradient across the inner mitochondrial membranes.

### 5.3. Taurine Regulates Intracellular Calcium Homeostasis

Several studies from El-Idrissi’s group have shown that taurine regulates intracellular calcium homeostasis and protects against glutamate-induced mitochondrial damage and cell death [164,165,166,167,168]. In general, glutamate increases the intracellular calcium level and causes the collapse of the mitochondrial membrane potential and induces cell death. However, when cultured cerebellar granule cells were pretreated with glutamate, taurine prevented an increase in the mitochondrial calcium level, prevented mitochondrial membrane depolarization and prevented an impairment in the mitochondrial function [166]. One of the functions of mitochondria is energy metabolism, which is regulated by calcium. In glutamate-induced excitotoxicity, a collapse in the mitochondrial membrane potential causes a depletion in the energy production, as measured by ATP levels, and promotes neuronal death. However, when neuronal cells were pretreated with taurine, the glutamate-induced excitotoxicity effects were inhibited, along with increased energy metabolism [165]. Meanwhile, additional studies [169,170,171,172] have shown taurine also regulates calcium homeostasis to maintain cardiac contractile function. In general, heart failure is caused by impaired contraction due to calcium mishandling, which reduces the calcium sensitivity of the contractile proteins, and insufficient ATP generation to drive contraction. Studies by Steele et al. [173] and Galler et al. [174] showed that physiological concentrations of taurine increase the calcium sensitivity of contractile proteins and modulate cardiac contractility. As calcium is known to regulate mitochondrial oxidative phosphorylation to produce ATP [175], regulation of intracellular calcium homeostasis by taurine can improve the energy production through maintenance of mitochondrial function.

### 5.4. Taurine Inhibits Mitochondria-Mediated Apoptosis

It has been thought that taurine acts at the level of mitochondria to inhibit apoptosis. Indeed, Jong et al. [135] showed that TauTKO hearts pretreated with mitotempo, a mitochondria-targeted antioxidant, were protected against oxidative stress and mitochondrial apoptosis. Interestingly, Takatani et al. [79] showed that taurine pretreatment does not prevent the release of cytochrome c and the reduction in mitochondria membrane potential during ischemia. However, taurine pretreatment prevents ischemia-induced cleavage of caspase 9 and caspase 3 [79]. Generally in apoptosis signaling, cytochrome c, Apaf-1 and caspase 9 form a complex known as apoptosome, which activates caspase 9 and the subsequent downstream caspase-3-mediated signaling cascade [176]. In another study by Leon et al. [177], it was shown that taurine protects against glutamate-induced apoptosis by inhibiting glutamate-induced membrane depolarization, potentially by acting on the chloride channels and preventing excessive calcium influx. As a result, glutamate-induced activation of calpain is inhibited, which then prevents Bax translocation to the mitochondria and the subsequent cytochrome c release. Similar mechanisms have also been reported by Wu and Prentice [178,179]. Similarly, Taranukhin et al. [180] showed that taurine prevents ethanol-induced mitochondria-mediated apoptosis by increasing the levels of Bcl-2. Restoration of Bax:Bcl-2 levels prevents Bax translocation to the mitochondria, and thus protects against activation of the mitochondria-mediated apoptosis.

## 6. Clinical Application of Taurine in Mitochondria-Targeted Pathologies

Several clinical studies investigating the therapeutic potential of taurine have been reported, particularly in relation to its role as an antioxidant in improving mitochondrial function. Below, we report several known clinical studies on taurine supplementation in various pathologies in humans.

### 6.1. Cardiovascular Diseases

Taurine was first used to treat heart failure patients by Azuma’s group in Japan [29,30,181]. Most heart failure patients receiving taurine supplementation (2–3 g taurine daily for a range of four to eight weeks) showed improvement in the systolic left ventricular function, as evidenced by increased cardiac output and stroke volume, ejection fraction and mean velocity of circumferential fiber shortening. When comparing the effects of taurine to coenzyme Q_10_, no significant improvement in the systolic left ventricular function was observed [181]. Coenzyme Q_10_ is an antioxidant that mediates the transfer of electrons from complex I and complex II to complex III [42]. In heart failure patients, the levels of coenzyme Q_10_ were reduced and supplementation with coenzyme Q_10_ was shown to improve the symptoms among heart failure patients [182,183,184]. The effectiveness of taurine, but not coenzyme Q_10_, in improving myocardial energy in heart failure suggests the significant effect of taurine as an antioxidant, potentially by improving the myocardial energy production. In heart failure, a disturbance in ATP production affects myocardial contraction [185,186], thereby suggesting the significance of taurine in improving mitochondrial function through restoration of myocardial energy production. Similarly, another study conducted among heart failure patients in NYHA class II or III receiving 500 mg of taurine supplementation three times a day for two weeks showed improved exercise capacity [31]. Again, this study implicates the role of taurine as an antioxidant to improve mitochondrial function, potentially through restoration of energy production.

Taurine has also been supplemented among patients with hypertension. Taurine supplementation of 1.6 g per day for at least 12 weeks in prehypertensive patients or 6 g per day for 7 days in hypertensive patients lowered blood pressure and improved vascular function [187,188]. In addition, worldwide epidemiological studies among 61 different population groups in 25 different countries have reported that regular taurine intake through daily consumption of seafood, nuts, soy and milk reduces the prevalence of cardiovascular diseases that include hypertension and hypercholesterolemia [94,189,190,191,192]. While the underlying mechanisms by which taurine attenuates hypertension remain unclear, several in vitro and in vivo studies have suggested the antioxidant activity of taurine in reducing hypertension, which include reducing ROS generation [193,194], improving ATP production [195] and improving mitochondrial metabolism [196]. Indeed, hypertension has been largely associated with mitochondrial dysfunction, which includes mitochondrial oxidative stress [197], alteration of mitochondrial homeostasis and impaired energy production.

### 6.2. Metabolic Syndrome

Taurine supplementation among patients with type II diabetes mellitus also showed reduced oxidative stress and inflammation, as well as reduced diabetic complications, such as nephropathy, retinopathy and neuropathy [198,199,200]. In diabetic patients, plasma taurine concentrations have been reported to be reduced when compared to healthy control patients [201,202,203]. This reduction is associated with higher renal excretion of taurine and a lower intestinal absorption of taurine, suggesting a depletion in the bioavailability of taurine in diabetic patients [199,201,202,203]. Normal plasma taurine concentration has been reported to be about 44 μmol/L, while the normal whole blood taurine concentration is averaged to be 227 μmol/L in fasting humans [204]. Indeed, several in vitro and in vivo studies have also reported reduced taurine concentrations in diabetic models, which often are associated with impaired glucose tolerance, insulin resistance and impaired glucose and lipid metabolism [201,202,205,206,207]. It is well known that diabetic complications arise from hyperglycemia-induced oxidative stress, which usually originates from the mitochondrial respiratory chain as the primary source [208]. As a result of mitochondrial oxidative stress, the functions of the mitochondria are impaired, which are the main source of glucose and fatty acid metabolism. When taurine is supplemented, glucose levels are restored, insulin secretion is enhanced and glucose and lipid metabolism in the mitochondria are stimulated [209,210,211,212]. As an antioxidant, taurine potentially suppresses excessive oxidants generation in the mitochondria and maintains the functionality of mitochondria. While taurine is not known as a radical scavenger [151], the underlying mechanism of taurine as an antioxidant could potentially occur through conjugation with uridine of mitochondrial tRNA, equilibration of intracellular antioxidants or stabilization of mitochondrial homeostasis.

Despite the effectiveness of taurine in reducing diabetic complications in several clinical trials, there are a few studies contradicting the clinical effectiveness of taurine in diabetic patients. Firstly, Franconi et al. [202] reported that oral taurine supplementation of 1.5 g/day for 90 days in patients with insulin-dependent diabetes mellitus did not improve glucose metabolism, although plasma taurine concentration increased significantly in diabetic patients. Secondly, Chauncey et al. [213] reported that oral taurine supplementation of 3 g/day for 4 months among type 2 diabetic patients increased plasma taurine content but did not reduce the plasma glucose levels. Thirdly, Nakamura et al. [214] reported that oral taurine supplementation of 3 g/day for 12 months in patients with microalbuminuria of non-insulin-dependent diabetes mellitus increased plasma taurine content but did not reduce microalbuminuria, which is a strong marker of diabetic nephropathy.

Taurine has also been administered in overweight or obese individuals, which has shown promising therapeutic effects on lipid profiles. Rosa et al. [215] reported that obese women supplemented with 3 g/day of taurine for eight weeks had a significant increase in the content of both plasma taurine and adiponectin and a significant decrease in pro-inflammatory markers and the lipid peroxidation marker. In a separate study by Mizushima et al. [216], healthy individuals were given high-fat and high-cholesterol diets for three weeks. At the same time, when these individuals were also given 6 g/day of taurine for three weeks, the increase in the levels of total cholesterol and low density lipoprotein (LDL)-cholesterol was attenuated. De Carvalho et al. [217] recently showed that a combination of 3 g taurine supplementation and exercise training for eight weeks among obese women improved lipid metabolism and mitochondrial activity in the subcutaneous white adipose tissue. In addition, regular taurine intake through daily consumption of seafood, nuts, soy and milk has been shown to reduce the prevalence of metabolic syndrome, which includes obesity [94,189,190,191,192]. This observation is based on worldwide epidemiological studies conducted in more than 60 different populations around the world [218]. The antioxidant role of taurine in modulating the lipid profiles in obesity is likely to occur through maintenance of mitochondrial homeostasis and suppression of oxidative stress that stimulates lipid metabolism and subsequent restoration of energy production [215,216,217]. Indeed, this is consistent with several in vivo and in vitro studies on metabolic syndrome, showing the suppression of lipid peroxidation and restoration of mitochondrial function with taurine treatment [219,220,221,222,223].

### 6.3. Mitochondrial Diseases

Recent studies on mitochondrial diseases such as MELAS and MERRF revealed a reduction in the formation of taurine-modified uridine of mitochondrial tRNAs, specifically 5-taurinomethyluridine (τm^5^u) and 5-taurinomethyl-2-thiouridine (τm^5^s^2^u) [145,148,149]. When taurine was supplemented, Homma et al. [82] observed an increase in the taurine modification of the mitochondrial tRNA^Leu(UUR)^, as well as improvement of the mitochondrial function in the iPSC generated from a MELAS patient. Indeed, oral taurine supplementation of either 9 g or 12 g per day for 52 weeks in MELAS patients showed prevention of stroke-like episodes, as well as increased modification of taurine-conjugated uridine of mitochondrial tRNA^Leu(UUR)^ [27,28]. In a separate study by Fukuda and Nagao [224], plasma taurine content was reduced in one Japanese man diagnosed with MELAS and maternally inherited diabetes and deafness (MIDD). While the taurine-containing modification of mitochondrial tRNA was not examined in this study, it is plausible that the formation of taurine-modified uridine was absent due to low plasma taurine content. Therefore, it is likely that oral taurine supplementation in this MELAS/MIDD patient could potentially increase the plasma taurine content, enhance taurine-containing modification of uridine and improve his clinical symptoms. Indeed, the significance of the taurine-containing modification of uridine has been established in several in vitro and in vivo studies that demonstrated the efficiency of mitochondrial protein synthesis, improvement of mitochondrial respiratory activity, restoration of energy production, suppression of oxidative stress and maintenance of global protein homeostasis [27,28,82,145,148,149,224].

### 6.4. Neurological Disorders

Currently, there are no reported clinical studies on taurine supplementation among patients with neurodegenerative diseases. However, there have been some in vivo studies assessing the therapeutic effects of taurine in several mouse models of neurodegenerative diseases, which include Parkinson’s disease [225,226] and Alzheimer’s disease [227,228,229,230]. In these studies, it has been concluded that taurine suppresses pathological changes by maintaining the mitochondrial homeostasis. A common feature in neurodegenerative diseases is glutamate-mediated hyperexcitability that leads to calcium overload, collapse in mitochondrial membrane potential and increased ROS production [231,232,233,234,235,236]. As mitochondrial damage is commonly observed in neurodegenerative diseases [233,234,235], it is sensible to presume that the cellular damage is caused by the mitochondrial ROS production. Indeed, several studies investigating the underlying mechanisms of neurodegenerative diseases have reported a reduction in the respiratory chain activity, a decrease in the ATP production, a collapse in the mitochondrial membrane potential and an increase in mitochondrial ROS production [11,234,235,237,238]. As taurine is known to modulate the mitochondrial function, it is plausible that taurine supplementation would improve the clinical symptoms in patients with neurodegenerative diseases.

The clinical effectiveness of taurine in neurodevelopmental disorders has been reported in several studies by Erickson et al. [239,240,241,242,243]. These clinical studies used acamprosate, which is a synthetic taurine analogue. Acamprosate is approved by the United States Food and Drug Administration (FDA) to treat alcohol dependence [244]. In the first study, oral acamprosate supplementation of 1 g/day for 21 weeks in three adults with Fragile-X syndrome showed significant improvement in communication [240]. In the second study, oral acamprosate supplementation of 1 g/day for 10 weeks in twelve young children with Fragile-X syndrome and comorbid autism spectrum disorders showed significant improvement in social skills and inattention/hyperactivity [243]. Additional studies on oral acamprosate supplementation of 1 g/day for 20 weeks in young children with autism spectrum disorders showed significant improvement in social deficits [239,241,242]. Neurodevelopmental disorders are commonly characterized by excessive glutamatergic and deficient GABAergic neurotransmission, which is associated with social impairment [245,246,247]. Glutamate excitotoxicity can lead to calcium overload, collapse in mitochondrial membrane potential and increased mitochondrial ROS production, which can cause mitochondrial dysfunction [231,232,233,234,235,236]. Many studies have increasingly shown the link between neurodevelopmental disorders and mitochondrial dysfunction [248,249,250]. As acamprosate is an analogue of taurine, which is an antioxidant that maintains the mitochondrial homeostasis, it is plausible that acamprosate improves the clinical symptoms in neurodevelopmental disorders by maintaining the mitochondrial homeostasis.

## 7. Conclusions

Taurine is a simple but unique sulfur-containing amino acid that has multiple physiological functions, including the maintenance of mitochondria health. While taurine is widely known as an antioxidant, its underlying mechanism remains unclear as taurine is not a radical scavenger. Studies conducted by Suzuki and colleagues have shown that taurine conjugates with mitochondrial tRNA^Leu(UUR)^ or tRNA^Lys(UUU)^ for proper codon–anticodon interaction to facilitate synthesis of mitochondrial-encoded proteins. Inefficiency of taurine modification of mitochondrial tRNA promotes inefficient translation of mitochondrial proteins, which are components of the electron transport chain. As a result, the assembly and stability of the respiratory chain complexes are impaired, which causes a sluggish response in the electron flux, yielding superoxide production. Taurine also regulates intracellular calcium homeostasis and intracellular antioxidant activity and inhibits apoptosis. As evidenced in many in vitro, in vivo and clinical studies on taurine supplementation, the occurrence of mitochondrial dysfunction, reduced energy production, oxidative stress and apoptosis are mostly inhibited. Taurine therapy, therefore, could potentially improve mitochondrial health, particularly in mitochondria-targeted pathologies, such as cardiovascular diseases, metabolic diseases, mitochondrial diseases and neurological disorders. Whether the protective mechanism on mitochondria primarily relies on the taurine modification of mitochondrial tRNA requires further investigation.
